# Upper-lip laser frenectomy without infiltrated anaesthesia in a paediatric patient: a case report

**DOI:** 10.1186/1757-1626-2-7138

**Published:** 2009-05-20

**Authors:** Panagiotis Kafas, Christos Stavrianos, Waseem Jerjes, Tahwinder Upile, Michael Vourvachis, Marios Theodoridis, Irene Stavrianou

**Affiliations:** 1 Department of Oral Surgery, Surgical Implantology and Radiology, School of Dentistry, Aristotle UniversityAgiou Dimitriou Street, Thessalonica 541 24Greece; 2 Department of Endodontics, School of Dentistry, Aristotle UniversityThessalonica, 541 23Greece; 3 Unit of Oral and Maxillofacial Surgery, UCL Eastman Dental Institute256 Grays Inn Road, London WC1X 8LDUK; 4 Head and Neck Centre, University College London Hospital253 Euston Road, London, NW1 2BUUK; 5 Department of Surgery, University College SchoolGower Street, London WC1E 6BTUK; 6 Private DentistThessalonicaGreece

## Abstract

Labial frenectomy is a common surgical procedure in the field of oral surgery. Labial frenectomy is a procedure usually done for orthodontic reasons. The role of laser surgery in the oral cavity is well established. The use of diode laser frenectomy without infiltrated anaesthesia is currently under investigation. Needle-less oral surgery, without infiltrated anaesthesia, is a novel situation in paediatrics with paramount importance.

## Introduction

The role of laser in dentistry is well-established in conservative management of oral diseases [1]. In oral surgery, it is still under evaluation [2-4]. This seems to be unavoidable if we consider that in the surgical science it is very difficult to perform an organized double-blind randomized controlled trial, which is the prerequisite for the estimation of each surgical technique.

The diode light equipment may be considered a modern laser technology in the field of dentistry. Diode laser showed good results as an extra adjunct to the classical methods in the management of inflamed periodontal tissues and endodontics [3,5,6].

Currently, painless procedures are secured by using local or general anaesthesia. This case report describes the parameters of performing upper labial frenectomy in pediatrics without infiltrated local anaesthesia. Moreover, the reassurance of the patient about the painless procedure is one of the most important criteria.

## Case presentation

**Figure 1.**
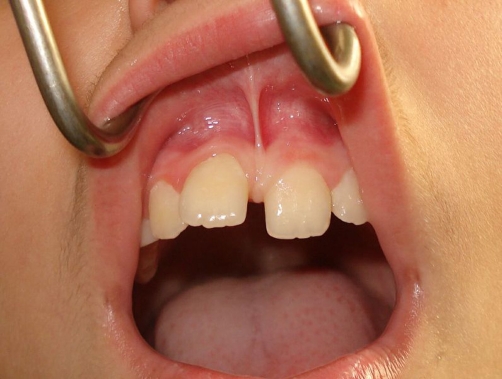
The upper anterior labial frenum is extended to the palatal inter-incisal area causing dental discrepancies.

A 9-year-old Caucasian boy of Greek nationality was referred by his orthodontist for assessment of the upper anterior labial frenum (Figure 1). The medical history was free. The dental history was composed of very minor dental procedures depending on the growth of the child. No allergies reported. The weight of the patient was 32 kg. The height of the patient was 112 cm.

The clinical examination revealed the presence of a high frenum attachment pathologically extending to the palatal inter-incisal region. As a consequence this presented a pathological eruption of the upper central incisors laterally, and towards to the canines.

**Figure 2.**
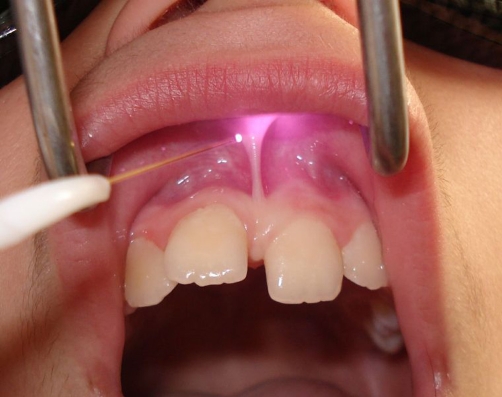
The fiber-optic of diode laser applied on the labial area first, using specific pain-free parameters.

It was decided to perform laser frenectomy without infiltrated anaesthesia using specific laser parameters (Figure 2). The laser equipment was defined by the manufacturer (Lamda Scientifica Srl) as a class-II B device according to the CE conformity statement, and the parameters were 1400 mW at 808 nm with continuous output. The optical fiber used was 300 μm diameter allowing a very fine soft tissue cut. According to the manufacturer, the optical protective glasses had an Optical Density > 5 at the wavelength of emission from the diode. According to standard EN 60825 CEI 76-2 II, the minimum optical density has been estimated to be 4.96 at 0.05 m.

**Figure 3.**
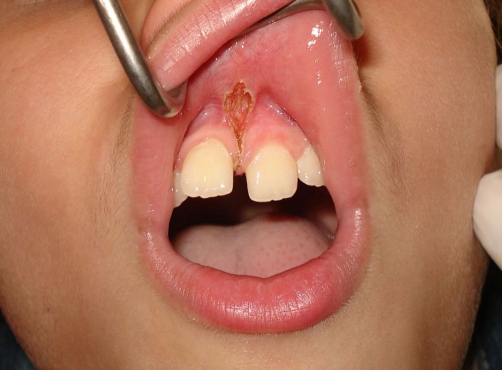
The final rhomboidal laser cut did not require sutures or periodontal dressing. Postoperative haemostasis is optimum.

**Figure 4.**
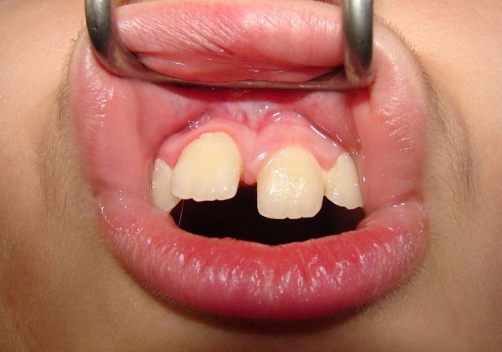
Ten days later, the healing of wound was assessed and described as optimal. Notice the biofilm on teeth surface due to inadequate oral hygiene.

The labial frenum was sprayed with lidocaine twice. The laser fiber was applied vertically and laterally to the frenum initially causing disruption of the mucosa continuity. This easily allowed performing a deeper cut of the frenum in a horizontal dimension. The design of the frenectomy was rhomboidal allowing easy pass of the fiber-optic between the central incisors and from the buccal to palatal area. The whole procedure was performed in about five minutes, without pain. No sutures were required. Haemostasis was optimum immediately after the procedure (Figure 3). The patient was comfortable with no pain, either intra-operatively or post-operatively. The patient described the procedure as totally painless. Ten days later the healing was found to be uneventful (Figure 4). Pre-operatively, the parents of the patient had been informed verbally about the procedure finally signing a written consent form.

## Discussion

Frenectomy is a common procedure in the field of oral and maxillofacial surgery. The advantage of laser surgery includes higher precision when compared to surgical tools, which results in less pain, bleeding, swelling and scarring. The procedure is no time consuming, easy to perform in an outpatient set and no sutures are required, which decreases the risk of post-operative infection [7].

This case report described the advantages of diode laser surgery purposely omitting routine procedure as laser transmits energy to the cells causing warming, welding, coagulation, protein denaturization, drying, vaporization and carbonization [8].

The great advantage of diode laser frenectomy in paediatrics should be the avoidance of needle-infiltrated anaesthesia. Considering that children are more pain sensitive, this case report discussed a case of a child that had no external stimulus (laser) to react, which means that diode laser may be used under specific parameters safely in all age groups without infiltrated anaesthesia.

The main disadvantage is the time required for frenum excision by using diode laser in pain-free parameters if compared to electrosurgery and blade incision, which always requires anaesthesia. In severe cases of highly attached frenum the need of anaesthesia is essential. A critical thinking is required to explain which procedure is medically superior to others. We suggest the use of diode laser, even if the clinician needs more time to complete the procedure. In our opinion, it is more important to avoid any painful needle injection in mild and moderate frenum attachment.

Pain is an unpleasant sensory and emotional experience associated with actual or potential tissue damage [9]. Currently, a way of avoiding such an experience in paediatric is to perform laser labial frenectomy without infiltrated anaesthesia. Clinicians know that children very much fear needle injections [10]. Any previous painful experience affects the emotional status of the children when a dental procedure is to be performed.

Concluding, pain is a subjective feeling, which is very difficult to be assessed. Pain perception is another important issue in creating guidelines for surgical procedures. Diode laser surgery may be considered a useful tool for the clinician in performing paediatric labial frenectomy. The need for a randomized controlled trial is emphasized in order to establish the exact efficacy of this technique if compared to other methods. It is obvious that diode laser frenectomy may be performed without infiltrated anasethesia with the optimum healing post-surgically. In severe cases of soft tissue excision the need of anaesthesia may be essential [11].
